# Sun-aged red date vinegar-based beverage: integrated analysis of fermentation, sensory, volatile, and bioactive properties

**DOI:** 10.1016/j.fochx.2025.103023

**Published:** 2025-09-12

**Authors:** Zeshan Ali, Zinanone Rosaire Brottier, Jameel M. Al-Khayri, Rana Adnan Tahir, Sam Al-Dalali, Othman Al-Dossary, Bader Alsubaie, Mustafa I. Almaghasla

**Affiliations:** a College of Food Science and Technology, Bohai University; National & Local Joint Engineering Research Center of Storage, Processing and Safety Control Technology for Fresh Agricultural and Aquatic Products, Jinzhou, Liaoning 121013, China; b Department of Agricultural Biotechnology, College of Agriculture and Food Sciences, King Faisal University, Al-Ahsa 31982, Saudi Arabia; c Department of Biology, Sultan Qaboos University, Muscat 123, Oman; d School of Food and Health, Guilin Tourism University, Guilin 541006, China; e Department of Food Science and Technology, Faculty of Agriculture and Food Science, Ibb University, Ibb 70270, Yemen; f Plant Pests and Diseases Unit, College of Agriculture and Food Sciences, King Faisal University, Al-Ahsa 31982, Saudi Arabia

**Keywords:** Red date vinegar, Fermentation, Volatile compounds, Sensory evaluation, Molecular docking, Functional beverage

## Abstract

This study developed a functional vinegar-based beverage using red date (*Ziziphus jujuba* Mill.) vinegar produced through controlled fermentation and traditional sun-aging. Alcoholic and acetic acid fermentations, followed by four months of sun-aging, yielded a vinegar beverage characterized by diverse volatile compounds and notable antioxidant activity. To improve flavor and reduce sourness, goji berry juice and honey were incorporated. Formulations were assessed for pH (3.35–3.65), total soluble solids (3–7°Brix), antioxidant activity (DPPH, ABTS), and volatile profiles. Sensory analysis indicated moderated sourness and high flavor acceptability. Partial least squares regression showed a strong correlation (94 %) between E-tongue data and sensory scores. SPME–GC/MS identified key volatiles including ethyl acetate and oleic acid. Molecular docking predicted favorable interactions between 2,3-dihydro-1,1,3-trimethyl-3-phenyl-1H-indene and metabolic and cardiovascular targets. These results support the development of a palatable, bioactive functional vinegar-based beverage with potential health-promoting properties.

## Highlights

• A novel functional beverage was developed using sun-aged red date vinegar as a base.
• Post-fermentation addition of goji berry juice improved sensory quality and enriched volatile composition.
• GC–MS and electronic tongue analyses revealed strong correlations between chemical profiles and sensory attributes.
• *In silico* molecular docking suggested functional relevance of key aroma-active volatiles.

## 1 Introduction

Functional beverages derived from fermented fruits are gaining increasing attention due to their potential to deliver health-promoting phytochemicals that support metabolic and cardiovascular health (Gupta et al., 2023). Cardiovascular disease (CVD) and type 2 diabetes mellitus (T2DM) are leading global health burdens, often coexisting due to shared pathophysiological mechanisms such as oxidative stress, inflammation, insulin resistance, and endothelial dysfunction (Siam, Snigdha, Tabasumma, & Parvin, 2024). Dietary interventions rich in antioxidants and bioactives have shown promise in mitigating these risks.

Vinegar produced from fruits *via* controlled fermentation offers nutritional and sensory benefits, including improved shelf life, distinctive volatile profiles, and enhanced antioxidant capacity (Luzón-Quintana, Castro, & Durán-Guerrero, 2021). Among fruit substrates, *Ziziphus jujuba* (red date) is particularly attractive due to its high phenolic content, vitamins, and minerals, which contribute to its antioxidant and anti-inflammatory properties (Budak, 2022). However, the application of sun-aged red date vinegar as a base for functional beverage development remains underexplored, especially regarding sensory optimization and bioactive enhancement.

To balance the strong acidity of the vinegar and improve consumer acceptance, goji berry (*Lycium barbarum*) juice was added post-fermentation. This not only enhanced sweetness and aroma but also preserved the juice's native polyphenols and bioactives. Goji berries are widely recognized for their carotenoids, polysaccharides, and flavonoids associated with immune modulation, glucose regulation, and cardiovascular protection (Yang et al., 2022).

Supporting this approach, previous studies have shown that combining goji berries with red dates or their derivatives can enhance sensory attributes and overall acceptability. Goh, Ng, and Nyam (2021) found that infusing goji berries and red dates improved the flavor, aroma, and overall quality of kenaf leaves tea. Likewise, incorporating *Lycium barbarum* and jujube polysaccharides into goat milk cheese enhanced texture, water retention, and structural integrity, contributing to improved product appeal (Wang et al., 2023).

In addition to physicochemical and sensory evaluation, this study incorporated molecular docking to screen major volatile compounds for potential interactions with key proteins involved in metabolic and cardiovascular regulation, such as angiotensin-converting enzyme (ACE), sodium-glucose cotransporter 1 (SGLT1), and mitogen-activated protein kinase p38 (MAPK p38). This bioinformatics approach complements experimental analysis by predicting potential interaction patterns and energy-based suitability of food-derived compounds with their target proteins, thereby offering preliminary insights that require further validation through *in vitro* or *in vivo* studies (Auwal, Zainal Abidin, Zarei, Tan, & Saari, 2019).

Therefore, this study aimed to develop a novel vinegar-based functional beverage using sun-aged red date vinegar as the core ingredient. To enhance flavor balance, sweetness, and bioactive composition, goji berry juice and honey were incorporated into the formulation. Comprehensive analyses—including physicochemical profiling, phytochemical and antioxidant assays, sensory evaluation, volatile compound characterization, and *in silico* molecular docking—were conducted to identify the optimal formulation and support the development of a consumer-acceptable beverage with potential health-promoting properties.

## 2 Materials and methods

### 2.1 Ingredients

Dates were obtained from the market in Xi'an, China. Dried goji berry fruits were sourced from Ningxia, Northern China. Honey was procured from the Matsuyama New District Cold Honey Shop in the Jinzhou local market, China.

### 2.2 Chemicals and strains

The following chemicals were used in this study: 2,2-diphenyl-1-picrylhydrazyl (DPPH), methanol (MeOH), sodium carbonate (Na₂CO₃), Folin-Ciocalteu's phenol reagent, sodium hydroxide (NaOH), aluminum chloride (AlCl₃), ethanol, and sodium nitrite (NaNO₂), all of which were purchased from Shanghai Macklin Biochemical Co., Ltd. and were of analytical grade. Microbial strains *Saccharomyces cerevisiae* (Angel Yeast Co., Ltd., China) and Acetobacter species (Baoji Dingli Biotechnology Co., Ltd., China) were employed for fermentation processes. These strains were selected for their high fermentation efficiency, stable metabolic activity, and ability to produce the desired metabolites under controlled conditions, which were in line with standard quality criteria.

### 2.3 Production of red date vinegar

Red date vinegar was prepared following the same protocol as our previous study (Ali et al., 2025). Briefly, sorghum and wheat bran (1:1 *w*/w) were ground (20-mesh), mixed with water (1:3 *w*/*v*), and steamed at 100 °C for 2 h. After cooling to 35–40 °C, pitted red dates (1:3 w/w) and Daqu **(**a traditional Chinese fermentation starter made from grains and mixed microbial cultures**)** (62.5 % w/w) were added. *Saccharomyces cerevisiae* (0.5 % w/w) and *Acetobacter spp.* (3 % *v*/v) were used for sequential alcoholic (7–10 days, 25–30 °C) and acetic acid fermentation (10–14 days, 30–35 °C). The vinegar was then filtered, pasteurized (85 °C, 10 min), and sun-aged in jars for 4 months. During sun-aging, jars were placed outdoors under natural sunlight, with daily temperature fluctuations of 25–35 °C and relative humidity ranging from 60 to 75 %. Sunlight intensity was typically 30–60 klux during daytime. The jars were loosely covered with breathable cloth to prevent contamination while allowing air exchange, consistent with traditional sun-aging practices.

### 2.4 Preparation of goji berry juice

Dried goji berries (50 g) were rinsed under tap water and soaked for approximately 30 min to soften. After washing, the berries were drained and blended with 4 cups of mineral water (approximately 1L) until smooth. The combined mixture was then strained to extract as much juice as possible, yielding approximately 150–180 mL of juice, which was used to prepare the different beverage samples.

### 2.5 Formulation of beverage samples

Six beverage samples were prepared by incorporating goji berry juice into the sun-aged red date vinegar base. Each 100 mL beverage formulation contained red date vinegar, goji berry juice, honey, and water in the following proportions (mL/mL/g/mL): A (6/4/4/86), B (7/5/5/83), C (8/6/5/81), D (10/8/6/76), E (12/10/6/72), and F (14/10/9/67). Ingredient ratios were determined through preliminary formulation trials, with vinegar kept greater than goji juice in all samples to preserve classification as a vinegar-based beverage. Honey was proportionally adjusted to balance sweetness against acidity, while incremental changes in vinegar and goji juice created a stepwise gradient in acidity, sweetness, and color intensity for systematic evaluation of sensory thresholds and consumer acceptability ranges. Goji berry juice was added post-fermentation to moderate acidity, enhance color and aroma, and retain its phytochemical content, while final optimization was guided by sensory evaluation using a trained panel (Pauline, Alexandre, Andoseh, Abeline, & Agatha, 2017).

### 2.6 Physical-chemical analysis of functional beverage

#### 2.6.1 pH

pH was measured using a pH meter (InoLab WTW Series 730, China) equipped with a glass electrode. Before measurements, the instrument underwent multi-point calibration with standard buffer solutions, using calibration points encompassing the expected sample pH range, per the manufacturer's instructions. Measurements were performed at 25 °C.

#### 2.6.2 Total soluble solids

Total soluble solids (TSS) were measured using a portable refractometer (Shanghai Lichen Bangxi Instrument Technology Co., Ltd) equipped with a percent scale. The results were expressed as percent soluble solids (°Brix). Calibration against sucrose solutions of known concentration gave a coefficient of determination of R^2^ = 0.8816. All measurements were performed in triplicate.

#### 2.6.3 Ascorbic acid content

Vitamin C content was determined by titration with 0.04 % 2,6-dichlorophenol indophenol dye solution, following minor modifications to standard procedures. A 5 mL beverage sample was mixed with 15 mL of 0.4 % oxalic acid and titrated until a persistent pink endpoint was observed. The concentration of ascorbic acid was calculated using the formula:

$$\mathrm{Ascorbic acid}\left(\mathrm{mg}/100\mathrm{mL}\right)=\frac{C\times V\times 100}{S}$$

where C is the ascorbic acid equivalence of 1 mL of titrant (mg/mL, determined from the standard curve), V is the titrant volume consumed in the titration (mL), and S is the sample aliquot titrated (mL). All measurements were conducted in triplicate. This approach is consistent with the calculation principle applied in fruit juice analysis by Devolli, Stafasani, Shahinasi, Dara, and Hamiti (2021).

#### 2.6.4 Total carbohydrate content

The total carbohydrate content was determined using the phenol‑sulfuric acid method, as described by Dubois, Gilles, Hamilton, Rebers, and Smith (1951) with slight modifications. This colorimetric method is commonly used to quantify soluble carbohydrates (monosaccharides, disaccharides, and oligosaccharides) in food matrices. Briefly, 2 mL of each beverage sample was mixed with 1 mL of 4 % phenol solution and 5 mL of 95 % sulfuric acid. The mixture was incubated at room temperature for 10 min, and absorbance was measured at 490 nm using a UV-2550 spectrophotometer (Shimadzu, Japan). A d-glucose standard curve was used for quantification, and results were expressed as mg of glucose equivalents per 100 mL of sample. All measurements were performed in triplicate.

### 2.7 Phytochemical analysis

#### 2.7.1 Total phenolic content

Total phenolic content (TPC) was determined according to the Folin-Ciocalteu reagent method, as defined by Singleton, Orthofer, and Lamuela-Raventós (1999), with slight adjustments. A sample of 5 mL was mixed with 1 mL of 80 % ethanol and 0.3 mL of Folin-Ciocalteu reagent. The mixture was allowed to react for 5 min, after which 10 mL of 7 % sodium carbonate solution was added and mixed thoroughly. Afterward, the mixture was kept at room temperature for 2 h to stand. All experiments were repeated three times, and the absorbance was measured at 740 nm *via* a UV-2550 spectrophotometer (Shimadzu, Japan), with the reagents used as the blank. The TPC was calculated as gallic acid equivalents (mg GAE/mL), and a calibration curve was prepared with a linearity range of 50–1000 μg/mL (R^2^ = 0.99).

#### 2.7.2 Total flavonoid content

The total flavonoid content (TFC) was ascertained based on a modified version of the method defined by Heimler, Vignolini, Dini, and Romani (2005). In this regard, 0.25 mL of the beverage sample was mixed with 1.25 mL of distilled water, followed by 75 μL of 5 % NaNO₂ solution. After 6 min, 150 μL of 10 % AlCl₃·6H₂O solution was added and allowed to stand for 5 min. Afterward, 0.5 mL of 1 M NaOH was added to the mixture, followed by distilled water until the volume reached 2.5 mL. The solution was mixed thoroughly, and the absorbance was measured at 510 nm against distilled water as a blank. The results were presented as milligrams of rutin (mg Ru/mL) using a calibration curve of rutin, with a linearity range of 10–1000 μg/mL (R^2^ = 0.99). It should be mentioned that all experiments were repeated three times.

#### 2.7.3 Total carotenoid content

Total carotenoid content (TCC) was determined using a modified protocol based on Sanusi and Adebiyi (2009). A 0.5 mL aliquot of the beverage was extracted in triplicate with 5 mL ethanol/BHT (100:1, *v*/*w*), vortexed, and incubated at 85 °C for 5 min. Saponification was achieved by adding 0.5 mL of 80 % KOH, followed by another 10 min incubation at 85 °C. After cooling in an ice bath with 3 mL deionized water, 3 mL n-hexane was added. Phase separation was performed by centrifugation at 2500 ×*g* for 5 min. The yellow upper hexane layer was collected and re-extracted four times until colorless (total 12 mL of hexane). A spectrophotometer measured the absorption at 450 and 503 nm against a hexane blank. TCC (μg/mL) was calculated using the equation:

$$\mathrm{Total Carotene}\;\left(\mathrm{\mu g}/\mathrm{mL}\right)=\left(4.642\times A₄₅₀\right)-\left(3.091\times A_{503}\right)$$

### 2.8 Determination of antioxidant activity

#### 2.8.1 DPPH (2,2-diphenyl-1-picrylhydrazyl) radical-scavenging activity

The DPPH radical-scavenging activity of the beverage was assessed following the method of Chen and Ho (1995), with minor modifications. Briefly, 0.3 mL of the sample was mixed with 3.8 mL of 0.1 mM DPPH in ethanol, vortexed for 1 min, and kept in the dark for 30 min. Absorbance was measured at 517 nm using a UV-2550 spectrophotometer (Shimadzu, Japan) against an ethanol blank. Antioxidant activity was expressed as mg Trolox equivalents per mL (mg TE/mL) based on a Trolox calibration curve (20–1000 μM, R^2^ = 0.99). All tests were performed in triplicate.

#### 2.8.2 ABTS radical-scavenging activity

ABTS radical-scavenging activity was determined using a modified method of Re et al. (1999). ABTS^+^ was generated by reacting 7 mM ABTS with 2.45 mM potassium persulfate and incubating in the dark at room temperature for 12–16 h. The solution's absorbance was adjusted to 0.7 with ethanol. For analysis, 0.3 mL of the sample was mixed with 0.3 mL of ABTS^+^ and incubated for 10 min at room temperature. Absorbance was measured at 734 nm using a UV-2550 spectrophotometer (Shimadzu, Japan). Antioxidant activity was expressed as mg Trolox equivalents per mL (mg TE/mL), based on a Trolox calibration curve (20–1000 μM, R^2^ = 0.99). All tests were conducted in triplicate.

### 2.9 Sensory and flavor analyses

#### 2.9.1 Ethical approval and consent

The sensory evaluation in this study involved adult human volunteers and was conducted in accordance with established ethical protocols for sensory research. As the study involved low-risk, commercially available food products (red date vinegar and goji berry juice) that are commonly consumed in China, and did not collect any personal or sensitive data, no formal ethics committee approval was sought. Nonetheless, all participants were thoroughly informed about the study's purpose, procedures, and their rights, including the right to decline or withdraw at any time without consequence. Written informed consent was obtained from all participants. No vulnerable populations were involved, and participant confidentiality was maintained throughout. The study complied with the ethical principles of the Declaration of Helsinki (World Medical Association, 2013) and established standards for sensory evaluation of food (Lawless & Heymann, 2010).

#### 2.9.2 Descriptive and hedonic sensory evaluation

Sensory evaluation was conducted at Bohai University (China) in two phases: a descriptive analysis by trained panelists and experts, and a consumer acceptance test. In the descriptive phase, 15 trained panelists and 5 sensory experts evaluated six beverage formulations using a modified quantitative descriptive analysis (QDA) method, adapted from Tobin, Moane, and Larkin (2013). Trained panelists were instructed in standardized protocols and assessed key sensory attributes, including sweetness, aroma, and sourness. Expert judges monitored evaluation consistency and protocol adherence.

Evaluations were conducted in an air-conditioned, uniformly lit room, with panelists seated at least 1 m apart. Sensory descriptors were established through a preliminary focus group, and the six samples were coded (A–F) and presented in randomized order across seven consecutive days, using freshly prepared samples each day.

For the consumer test, 30 untrained participants assessed six samples using a 9-point hedonic scale (1 = dislike extremely; 9 = like extremely) to rate appearance, taste, texture, aroma, and overall acceptability. Each consumer received 100 mL of each sample, presented simultaneously in random order. Following the evaluation, participants completed a short interview regarding product preferences and general beverage consumption habits.

#### 2.9.3 Electronic tongue (E-tongue) analysis

Instrumental taste profiling was performed using an E-tongue system with multiple taste sensors simulating human gustatory receptors (Yu, Zhang, Zhao, & Tian, 2018). Each sample was analyzed in triplicate. Prior to analysis, the system was calibrated using standard solutions representing primary taste modalities.

The E-tongue measured seven taste dimensions: bitterness, sourness, saltiness, umami, richness, astringency, and aftertaste (A/B). Electrical potential responses were recorded and processed using pattern recognition algorithms. Radar plots were generated to visualize taste profiles, and data normalization was applied to correct baseline drift. Partial least squares regression (PLSR) was used to evaluate the correlation between sensory panel scores and E-tongue outputs, facilitating objective validation of taste characteristics in relation to industrial formulation standards.

#### 2.9.4 Volatile compound profiling by SPME-GC–MS

Volatile compounds in the beverage samples were analyzed using solid-phase microextraction coupled with gas chromatography–mass spectrometry (SPME-GC–MS). A 5 mL sample was transferred into a 20 mL headspace vial containing 20 μL of cyclohexanone (1.0 μg/mL, internal standard) and sealed with a PTFE/silicone septum. A preconditioned DVB/CAR/PDMS fiber (50/30 μm) was exposed to the headspace at 55 °C for 5 min with magnetic stirring (equilibration), followed by extraction for 25 min. The fiber was then desorbed in the GC injector at 250 °C (splitless mode, 5 min). Between samples, the fiber was conditioned at 250 °C to prevent carryover.

GC–MS analysis used an HP-5MS capillary column (30 m × 0.25 mm i.d., 0.25 μm film thickness). The oven temperature was programmed as follows: initial temperature of 40 °C (held for 5 min), ramped to 120 °C at 4 °C·min^−1^, then to 250 °C at 10 °C·min^−1^ (held for 6 min). Helium was used as the carrier gas at 1.2 mL·min^−1^.

The MS was operated in electron ionization (EI) mode at 70 eV. The ion source and interface temperatures were set at 230 °C and 280 °C, respectively. Compounds were identified by matching mass spectra and retention indices with NIST 11 and Wiley 7.0 libraries entries. Retention indices were determined using a standard *n*-alkane series (C8–C20) analyzed under identical conditions.

All measurements were performed in triplicate. Semi-quantification was performed using the internal standard method, and concentrations of volatiles were calculated using the following equation:

$$\mathrm{Semi}-\mathrm{quantitation}=\frac{\mathrm{Peak area ratio}\;\left(\frac{\mathrm{volatile}}{\mathrm{IS}}\right)\times \mathrm{con}.\text{of IS}}{\mathrm{Sample weight}}\times 1000$$

### 2.10 *In silico* analysis of bioactive volatiles *via* molecular docking

Thirty-three major volatile compounds identified in sample F were subjected to molecular docking to evaluate their potential interaction with cardiovascular and metabolic protein targets. Chemical structures were retrieved from ChemicalBook (https://www.chemicalbook.com) and energy-minimized using the Molecular Operating Environment (MoE, 2024).

Target proteins were selected based on their documented relevance to CVD and T2DM: angiotensin-converting enzyme (ACE; PDB ID: 1O8A) (Zeng et al., 2025), p38 mitogen-activated protein kinase (MAPK p38; PDB ID: 4DLI) (Fan, Dong, Mu, & Liu, 2022), insulin receptor (IR; PDB ID: 1IR3) (Soliman & Schooling, 2022), and sodium-glucose cotransporter 1 (SGLT1; PDB ID: 3DH4) (Lin et al., 2021). Crystal structures were obtained from the Protein Data Bank.

Protein structures were prepared using UCSF Chimera 1.17.3 (Pettersen et al., 2004), by removing non-standard ligands, water molecules, and extraneous chains and adding polar hydrogens and Gasteiger charges.

Docking simulations were performed using AutoDock Vina (Trott & Olson, 2010), with ligand and protein structures converted to pdbqt format. According to prior docking protocols, grid boxes were defined around active site regions. The docking was executed using default exhaustiveness, and 15 binding poses were generated per ligand-target interaction. Binding affinity was evaluated based on the lowest binding energy (kcal/mol). Top-ranked ligand-protein complexes were visualized using MoE and UCSF Chimera (Pettersen et al., 2004).

### 2.11 ADMET analysis of bioactive volatiles

ADMET (Absorption, Distribution, Metabolism, Excretion, and Toxicity) properties of the top-ranked ligands were evaluated using the ADMETlab 2.0 platform (https://admetmesh.scbdd.com/). This platform provided predictions of key pharmacokinetic parameters for each compound, including solubility, permeability, and potential toxicity. These properties were assessed to provide a comprehensive view of the bioactive volatiles' suitability for drug development. The ADMET analysis allowed for an initial screening of the compounds to ensure they possess favorable pharmacokinetic properties before further experimental validation.

### 2.12 Statistical analysis

Results are expressed as mean values. One-way ANOVA followed by Tukey's post-hoc test and descriptive and correlation analyses were used for parametric variables. Partial Least Squares Regression (PLSR) was applied to examine correlations between electronic tongue (E-tongue) responses and sensory scores. A *p*-value <0.05 was considered statistically significant. Data analysis was performed using Excel (2016), SPSS (29.0.2.0), and Origin (2021).

## 3 Results and discussion

This study aimed to develop a vinegar-based functional beverage by formulating sun-aged red date vinegar with goji berry juice and honey to enhance sensory properties and enrich bioactive composition. A series of formulations were prepared with varied ingredient ratios, ensuring the vinegar content was equal to or greater than the goji juice in all samples to maintain its identity as the fermented base. To guide product optimization, physicochemical (pH, total soluble solids), phytochemical (phenolics, polysaccharides), antioxidant (DPPH, ABTS), and sensory analyses were conducted. These evaluations assessed nutritional quality and determined how ingredient ratios influenced acidity, sweetness, aroma, and palatability. The final optimized sample (F) balanced flavor acceptability and functional potential, demonstrating the feasibility of combining fermented vinegar with complementary bioactive-rich ingredients for consumer-friendly functional beverages.

Red date vinegar was obtained *via* sequential fermentation, in which *Saccharomyces cerevisiae* converted sugars into ethanol, contributing esters and phenolic compounds to the flavor profile, while *Acetobacter* oxidized ethanol into acetic acid, improving acidity and preservation (Lynch, Zannini, Wilkinson, Daenen, & Arendt, 2019). Goji berry juice was prepared by rehydrating and blending dried fruits with mineral water, yielding an extract rich in vitamins, antioxidants, and polysaccharides. *Honey was incorporated as a natural sweetener to balance acidity and enhance total soluble solids, and it was also acknowledged as a potential source of phenolic compounds contributing to the overall antioxidant capacity of the beverage, although its specific effect was not measured independently.*

Fermentation and sun-aging contributed to increased bioavailability of nutrients and the development of complex volatile compounds (Pinto, Vilela, & Cosme, 2022). Incorporating these ingredients into a ready-to-drink beverage improved sensory acceptance and standardized composition, and it offered a convenient delivery format that enhances the potential for regular consumption, especially compared to vinegar alone. The final formulation resulted in a noticeable increase in pH, which reduced acidity and moderated the intense sourness typical of unblended vinegar, thereby enhancing palatability and supporting broader consumer acceptance.

Formulations were designed to meet safety guidelines limiting vinegar (acetic acid) inclusion to ≤20 % *v*/v in ready-to-drink beverages to avoid excessive acidity and potential gastrointestinal discomfort (Ali et al., 2016). All samples contained 6–14 % v/v red date vinegar, remaining well below this threshold. Goji berry juice (4–10 % *v*/v) enriched phytochemical content and aroma while reducing acidity. Honey (4–9 g) and water were adjusted to balance sweetness and total soluble solids for optimal sensory outcomes. Water adjustments were an inherent part of the formulation design to create gradients in acidity, sweetness, and flavor intensity, and were not considered a confounding factor in the sensory evaluation. This formulation approach ensured that the optimized sample (F) delivered desirable flavor complexity and functional benefits while remaining safe for regular consumption.

Chemical characterization and sensory evaluations confirmed the impact of goji berry and honey enrichment on overall flavor balance and acceptability. These findings support sun-aged red date vinegar as a base for functional beverages, combining health benefits with enhanced sensory properties and aligning with current functional food development trends.

### 3.1 Physicochemical properties

**Fig. 1:**
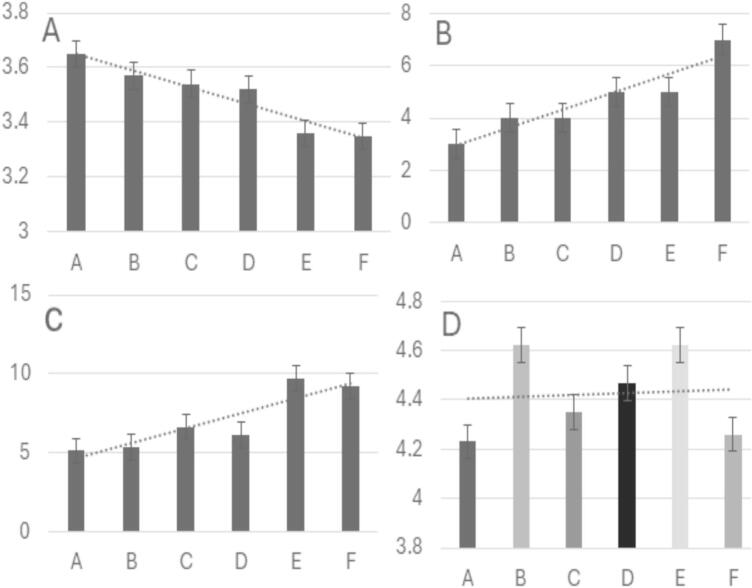
A–D. Physicochemical properties of a beverage across six samples: (A) pH values, (B) total soluble solids (°Brix), (C) ascorbic acid content (mg/100 mL), and (D) total carbohydrate content (g/100 mL). Columns with different patterns and superscript lowercase letters indicate significant differences between samples (*p* < 0.05).

Significant differences in physicochemical properties were observed among beverage samples **(**Fig. 1**).** pH values ranged from 3.35 (Sample F) to 3.65 (Sample A), reflecting differences in vinegar, honey, and water proportions. Sample F, which contained the highest honey content (9 g) and the lowest water volume (67 mL), exhibited the lowest pH, likely due to the acidity of honey (Seraglio et al., 2019) and the concentration of acidic components. In contrast, samples A and B, with lower vinegar and honey concentrations, showed higher pH levels. Buffering effects from goji berry juice and ingredient interactions may also have contributed to these variations (Pinto et al., 2022).

Total soluble solids (TSS) ranged from 3 to 7°Brix, with Sample F displaying the highest TSS due to increased honey addition. TSS, an important sweetness and flavor intensity indicator, strongly influences sensory perception and consumer acceptance. The balance between TSS and pH was critical in defining the flavor profile, as higher TSS can offset acidity and enhance palatability.

Ascorbic acid content varied from 5.11 mg/100 mL (Sample A) to 9.71 mg/100 mL (Sample E), correlating with the proportion of goji berry juice incorporated into the formulations. Higher ascorbic acid levels contribute to antioxidant potential and support health claims related to immune protection and metabolic benefits (Barrios Renteria, Espinoza-Espinoza, Valdiviezo-Marcelo, & Moreno-Quispe, 2022).

Carbohydrate concentrations remained relatively stable (4.23–4.62 g/100 mL) despite differences in the amounts of honey and juice. Consistency in carbohydrate content supports the maintenance of energy value and contributes to sensory characteristics such as mouthfeel and perceived sweetness (Gupta et al., 2023).

Overall, these findings highlight the role of formulation composition in determining pH, TSS, ascorbic acid, and carbohydrate levels—key parameters influencing product quality, nutritional value, and consumer preference. Future research could further explore the impact of these variables on sensory perception and potential health outcomes.

### 3.2 Phytochemical composition

Phytochemical analysis revealed notable variations among the beverage samples, including TPC, TFC, TCC, and antioxidant activity (assessed by ABTS and DPPH assays) **(**Table 1**).** These differences underscore the diversity of bioactive compounds across formulations, directly influencing their functional and nutritional properties.

**Table 1:** Phytochemical analysis of beverage samples.

Sample	TPC (mg GAE/mL)	TFC (mg Ru/mL)	TCC (μg/mL)	DPPH (mg TE/mL)	ABTS (mg TE/mL)
A	2.96 ± 0.04 ^a^	0.51 ± 0.01 ^a^	0.46 ± 0.03 ^a^	1.72 ± 0.16 ^a^	3.11 ± 0.60 ^a^
B	3.11 ± 0.06 ^ab^	0.54 ± 0.01 ^a^	0.63 ± 0.03 ^a^	0.88 ± 0.02 ^a^	2.90 ± 0.04 ^a^
C	3.29 ± 0.05 ^b^	0.98 ± 0.02 ^c^	0.57 ± 0.05 ^a^	0.75 ± 0.07 ^a^	3.99 ± 0.71 ^b^
D	3.45 ± 0.17 ^c^	0.78 ± 0.04 ^b^	1.13 ± 0.14 ^b^	0.73 ± 0.03 ^a^	3.14 ± 0.00 ^a^
E	3.34 ± 0.18 ^bc^	1.00 ± 0.03 ^c^	0.61 ± 0.02 ^a^	1.36 ± 0.35 ^a^	4.20 ± 0.41 ^b^
F	3.35 ± 0.19 ^bc^	0.90 ± 0.03 ^bc^	1.49 ± 0.01 ^c^	1.01 ± 0.00 ^a^	3.79 ± 0.02 ^b^

Note: Data are mean ± SD (*n* = 3). Different lowercase letters in a column indicate significant differences (*p* < 0.05, ANOVA, Tukey's test). TPC = Total Phenolic Content; TFC = Total Flavonoid Content; TCC = Total Carotenoid Content; DPPH = DPPH Scavenging; ABTS = ABTS Scavenging.

TPC ranged from 2.96 to 3.45 mg GAE/mL, consistent with values reported for red date vinegar (2.79–3.38 mg GAE/mL; Ali, Ma, Rashid, Wali, & Younas, 2019) and goji berry extracts (2.17–4.48 mg GAE/g; Islam, Yu, Badwal, & Xu, 2017). Sample F exhibited a notably high TPC (3.35 mg GAE/mL), suggesting strong antioxidant potential and suitability for functional applications. The relatively narrow range across samples indicates effective retention of phenolic compounds during formulation and processing.

TFC ranged from 0.51 to 1.00 mg Ru/mL, with samples C (0.98 mg Ru/mL) and E (1.00 mg Ru/mL) showing the highest levels. These exceeded values observed in our prior red-date vinegar study (0.67–0.97 mg/mL; Ali et al., 2019) and are comparable to those in stevia-based functional beverages (1.087 mg/mL; Martono & Soetjipto, 2014). Elevated flavonoid content, particularly in samples C and E, indicates enhanced antioxidant capacity. Although sample F had a slightly lower TFC (0.90 mg Ru/mL), its balanced composition contributed to superior sensory and functional performance.

TCC varied markedly, with sample F displaying the highest content (1.49 μg/mL), surpassing most samples and nearing the range reported for goji berries (1.51–1.96 μg/g; Islam et al., 2017) and carotenoid-rich vinegar (1.41 μg/mL; Kara, Assouguem, Zerhouni, & Bahhou, 2021). The higher TCC in sample F likely reflects the combined contribution of sun-aged red date vinegar and the incorporated goji berry juice, both known for their carotenoid richness.

Collectively, the high TPC, TFC, and TCC values confirm the antioxidant-rich profile of the enriched formulations**,** particularly sample F. These results underscore the potential of sun-aged red date vinegar as a functional beverage base that can be further enhanced through targeted ingredient incorporation to deliver nutritional benefits while maintaining acceptable sensory qualities.

### 3.3 Antioxidant activity

The antioxidant activity of the beverage samples, assessed *via* DPPH and ABTS assays, varied across formulations. Sample E exhibited the highest DPPH and ABTS radical scavenging activities at 1.65 mg TE/mL and 4.20 mg TE/mL, respectively, correlating with its elevated TPC and TFC. This strong association between phenolics and antioxidant capacity is consistent with existing literature (Sun & Shahrajabian, 2023). Samples F and C also demonstrated competitive antioxidant values, underscoring the role of phenolics, flavonoids, and carotenoids in enhancing functionality.

DPPH scavenging values for samples A–F were 1.83, 1.51, 1.39, 1.46, 1.65, and 1.47 mg TE/mL, respectively, with sample A showing relatively high activity, potentially due to a synergistic balance of bioactive compounds. ABTS results further supported the antioxidant trends, with sample C (3.99 mg TE/mL) closely following sample E.

Sample E's superior antioxidant performance can likely be attributed to its optimized ingredient ratio—containing less honey and higher levels of goji berry juice and sun-aged red date vinegar, both recognized sources of polyphenols. In contrast, the higher honey content in sample F may have contributed to a relative dilution of polyphenolic compounds, as sugars and other constituents with weaker free radical scavenging activity can lessen the apparent effectiveness of stronger antioxidants. Nevertheless, sample F, which ranked highest in sensory evaluation and GC–MS profiling, exhibited a substantial ABTS value of 3.79 mg TE/mL, making it a well-balanced candidate for functional beverage development.

Overall, variations in antioxidant potential across the formulations highlight the importance of polyphenol-rich ingredients. Despite ranking second in sensory evaluation, sample E showed the most robust antioxidant capacity, offering promise for oxidative stress mitigation. These findings support the development of natural, antioxidant-enriched beverages that align with consumer demand for health-promoting products, with antioxidant capacity assessed through widely accepted DPPH and ABTS assays. While these approaches adequately addressed the study objectives, future investigations may incorporate complementary assays such as ferric reducing antioxidant power (FRAP) or oxygen radical absorbance capacity (ORAC) to provide a more comprehensive antioxidant profile of sun-aged red date vinegar beverages.

### 3.4 Sensory analysis results

**Fig. 2:**
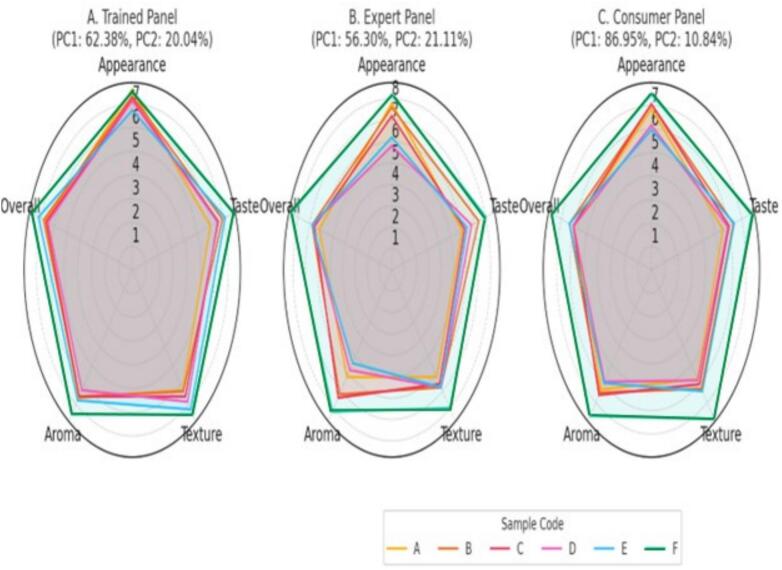
Radar plots with PCA scores for sensory evaluations of six beverage samples (A–F) by trained (A), expert (B), and consumer (C) panels. Sensory attributes include appearance, taste, texture, aroma, and overall acceptability. Color codes indicate sample identity, consistently applied across panels.

Sensory evaluation is essential for determining consumer acceptance, as it directly reflects how well the beverage appeals in terms of taste, texture, and overall enjoyment. As shown in the radar charts **(**Fig. 2**)**, sample F consistently achieved the highest mean scores in taste (7.80), texture (7.53), and overall acceptability (7.73). These favorable ratings are attributed to its balanced formulation, which included higher concentrations of goji berry juice (10 mL), sun-aged red date vinegar (14 mL), and honey (9 g). This combination enhanced the flavor profile by masking the acidity of the vinegar while adding sweetness and complexity. The improved texture likely resulted from the higher honey content, contributing to a smoother, more cohesive mouthfeel.

In contrast, samples A and D, which contained lower concentrations of red date vinegar (6–10 mL), goji berry juice (4–8 mL), and honey (4–6 g), exhibited comparatively milder sensory attributes. These ratings may reflect a less favorable balance of acidity, sweetness, and mouthfeel, underscoring the importance of optimizing ingredient ratios for sensory quality.

Principal component analysis (PCA) further supported these findings. In the trained panel, PC1 (62.38 %) and PC2 (20.04 %) explained 82.42 % of the total variance. Sample F emerged as the most preferred, especially for taste and overall liking, while samples A and D clustered together with lower sensory scores. Sample E stood out in aroma but scored lower on texture, indicating a potential trade-off between these attributes.

In the expert panel, PC1 (56.30 %) and PC2 (21.11 %) explained 77.41 % of the variance. Sample F again received high evaluations, particularly for taste and aroma, while sample E was noted for its aromatic intensity but rated lower in appearance and texture. Samples A and D maintained lower sensory performance, confirming consistency across panel assessments.

In the consumer panel, PC1 (86.95 %) and PC2 (10.84 %) captured 97.79 % of the variance, reflecting clear differentiation in preference. Sample F was rated highest across taste, aroma, and overall acceptability, while sample C scored well for texture. Samples A and D were again the least preferred, indicating cross-panel consistency in their lower appeal.

Overall, the radar charts and PCA plots confirm the superiority of sample F while highlighting the sensory limitations of samples A and D. These findings align with previous studies reporting that natural sweeteners and fruit extracts enhance the sensory appeal of functional beverages. For example, Saraiva, Carrascosa, Raheem, Ramos, and Raposo (2020) noted that fruit juice addition improves acceptability by balancing acidity and sweetness. Similarly, honey is known to enhance flavor complexity and overall preference.

In conclusion, sample F emerged as the most preferred formulation across all panels, balancing sensory appeal with health-promoting potential. While consumer and semi-trained panels emphasized overall flavor, expert feedback highlighted aroma–texture trade-offs. These insights can guide product refinement, particularly enhancing mouthfeel without compromising flavor complexity. Sample F's high sensory scores and phytochemical richness suggest strong market potential among health-conscious consumers.

#### 3.4.1 E-tongue taste profiling

E-tongues are invaluable in beverage analysis, offering a quantitative approach to measuring taste profiles and detecting subtle flavor differences that human taste testers may overlook. By employing E-tongue technology, manufacturers can conduct rigorous quality control and optimize product formulations based on consumer preferences, ultimately enhancing product acceptance in the market and providing a competitive edge.

**Fig. 3:**
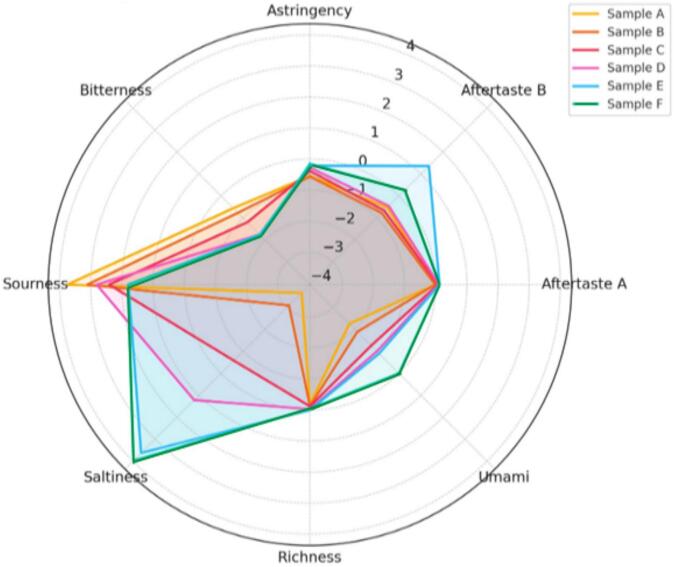
Radar plot showing the sensory profiles of six product samples (A–F) across attributes such as sourness, bitterness, astringency, aftertaste, umami, saltiness, and richness.

The E-tongue analysis revealed distinct sensory profiles among the beverage samples. Sample F exhibited the lowest bitterness (−1.82), the highest saltiness (3.98), and the most favorable aftertaste (0.13), making it particularly appealing to consumers **(**Fig. 3**).** The sensory evaluation results for sample F align with findings from Kuhar, Korošec, Bolha, Pravst, and Hristov (2020), indicating that consumers respond more favorably to food products characterized by enhanced saltiness and reduced bitterness. This observation supports the idea that optimizing these sensory attributes can significantly influence consumer acceptance. A key takeaway is that although consumers may not explicitly perceive subtle changes in saltiness intensity, an optimal salt level can positively impact their overall enjoyment. This leads to a favorable response to formulations that enhance taste profiles and reduce flavors like bitterness.

In contrast, sample A displayed the highest bitterness (−0.56) and sourness (3.80), indicating a more pungent and acidic taste profile that may be less desirable for some consumers. Sample E, on the other hand, showed milder sourness (1.79), neutral richness (0), and a strong aftertaste (1.36), suggesting a more balanced flavor profile that could cater to diverse consumer tastes. These results emphasize sample F's potential to fulfill consumer preferences for beverages with reduced bitterness and enhanced savory, salty notes.

**Fig. 4:**
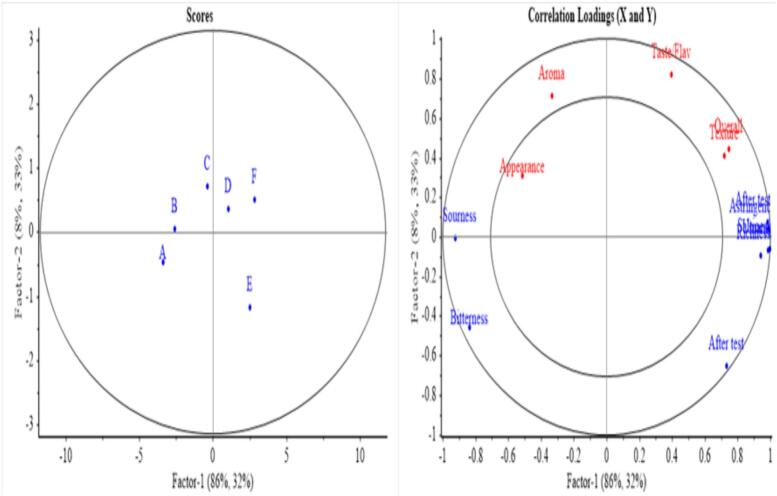
The PLSR analysis score plot (left) shows the sample distribution along Factor 1 and Factor 2, and the correlation loading plot (right) illustrates the relationship between sensory attributes and the main factors.

Following data normalization, partial least squares regression (PLSR) analysis was conducted to explore the relationship between E-tongue responses and sensory scores across all samples. The analysis revealed a distinct separation within the PLSR loading score diagram **(**Fig. 4**),** highlighting the unique characteristics of each formulation. Sample F was positioned on the right side of the diagram alongside samples D and E, indicating that it shares similar sensory attributes—mainly taste and texture, which likely contribute to its favorable reception among consumers. The clustering of samples D, E, and F suggests these formulations possess enhanced flavor profiles appealing to consumer expectations.

The analysis demonstrated that factors 1 and 2 accounted for 94 % of the total variation, confirming the robustness and fit of the model. Sample F exhibited positive correlations with key sensory attributes such as saltiness and richness, which are essential for achieving a well-balanced flavor profile. While samples A and B showed positive associations with aftertaste attributes, sample F's distinct sensory profile suggests it may minimize undesirable aftertaste effects commonly linked to bitterness or astringency. The observed separation in the PLSR analysis underscores the instrumental role of E-tongue data in differentiating beverage samples. Sample F's emphasis on attributes like saltiness and richness may further enhance overall acceptance and enjoyment among consumers.

The positive sensory evaluation scores and PLSR results suggest that sample F is well aligned with consumer expectations, reinforcing the importance of carefully balancing sensory attributes in beverage development. Thus, sample F's favorable positioning in the PLSR analysis highlights its potential as a leading formulation for consumer acceptance. These results emphasize the need to optimize flavor profiles to meet consumer demands effectively.

By integrating E-tongue analysis with traditional sensory evaluation, we gained more profound insights into how individual taste attributes—such as bitterness, saltiness, and aftertaste—contribute to overall consumer acceptance. Consequently, we selected the top-performing samples, F and E, for further volatile compound analysis, confirming their potential as marketable, health-promoting beverages with superior taste and flavor profiles.

### 3.5 Key aroma compounds and flavor profile insights

A volatile compound analysis was performed using SPME-GC–MS to evaluate aroma-contributing constituents across selected formulations. A total of 33 volatile compounds were identified in sample F, compared to 15 compounds in sample E **(**Table 2, Table 3**).** Representative GC–MS chromatograms supporting these identifications are provided as Supplementary Figs. S1 and S2 for samples E and F, respectively. These compounds spanned multiple chemical classes, including hydrocarbons, esters, ketones, alcohols, aldehydes, phenols, and acids, reflecting formulation-driven variation in aroma complexity.

**Table 2:** Profile of key volatile compounds in sample F.

Compound Name	CAS	Retention Index	Identification Methods⁎	Final Concentration (mg/L)
Ethyl Acetate	141-78-6	612	MS, RI	26.15 ± 1.31
1,3-Dimethyl-benzene	108-38-3	866	MS, RI	4.01 ± 0.20
p-Xylene	106-42-3	865	MS, RI	6.09 ± 0.30
1-Butanol, 3-methyl-, acetate	123-92-2	876	MS, RI	3.4 ± 0.17
2-Methyl-3-heptanone	13019-20-0	918	MS, RI	194.33 ± 9.72
4-Octanone	589-63-9	976	MS, RI	8.24 ± 0.41
1-Ethyl-3-methyl-benzene	620-14-4	987	MS, RI	5.64 ± 0.28
d-Limonene	5989-27-5	1018	MS, RI	6.15 ± 0.31
5-Nonanone	502-56-7	1073	MS, RI	12.31 ± 0.62
1-Butyl-1-methyl-2-propyl-cyclopropane	41977-34-8	1082	MS, RI	3.04 ± 0.15
Phenylethyl Alcohol	60-12-8	1116	MS, RI	6.01 ± 0.31
Pentyl-cyclohexane	4292-92-6	1135	MS, RI	33.31 ± 1.67
Octanoic acid, ethyl ester	106-32-1	1196	MS, RI	3.58 ± 0.18
Decanal	112-31-2	1206	MS, RI	5.21 ± 0.26
1-Phenyl-1-butanone	495-40-9	1263	MS, RI	3.2 ± 0.16
2,6,11-Trimethyl-dodecane	31295-56-4	1275	MS, RI	3.2 ± 0.16
(E)-5-Tetradecene	41446-66-6	1378	MS, RI	3.41 ± 0.17
Tetradecane	629-59-4	1400	MS, RI	28.71 ± 1.44
2,6-Bis(1,1-dimethylethyl)-2,5-cyclohexadiene-1,4-dione	719-22-2	1471	MS, RI	9.19 ± 0.46
Pentadecane	629-62-9	1500	MS, RI	29.25 ± 1.46
2-Cyclohexylidene-cyclohexanone	1011-12-7	1551	MS, RI	24.17 ± 1.21
Pentanoic acid, 2,2,4-trimethyl-3-carboxyisopropyl, isobutyl ester	1000140-77-5	1581	MS, RI	10.6 ± 0.53
Dodecanoic acid, ethyl ester	106-33-2	1595	MS, RI	11.56 ± 0.58
Hexadecane	544-76-3	1600	MS, RI	28.27 ± 1.41
2,6-Bis(1,1-dimethylethyl)-4-(1-oxopropyl)phenol	14035-34-8	1640	MS, RI	20.13 ± 1.01
2,3-Dihydro-1,1,3-trimethyl-3-phenyl-1H-indene	3910-35-8	1714	MS, RI	9.09 ± 0.45
Octadecane	593-45-3	1800	MS, RI	9.18 ± 0.46
Hexadecanoic acid, ethyl ester	628-97-7	1986	MS, RI	58.55 ± 2.93
Oleic Acid	112-80-1	2141	MS, RI	34.41 ± 1.72
Linoleic acid ethyl ester	544-35-4	2162	MS, RI	18.14 ± 0.91
2-Ethylhexyl trans-4-methoxycinnamate	83834-59-7	2339	MS, RI	17.35 ± 0.87
Methyl 8-[2-((2-[(2-ethylcyclopropyl)methyl]cyclopropyl)methyl)cyclopropyl]octanoate	10152-71-3	2350	MS, RI	17.3 ± 0.87
Bis(2-ethylhexyl) phthalate	117-81-7	2529	MS, RI	22.51 ± 1.13

⁎ Ms is the mass spectrum; RI is the retention index.

**Table 3:** Profile of Key Volatile Compounds in Sample E.

Compounds name	CAS	RI	Identification methods⁎	Final concentration (mg/L)
Ethyl Acetate	141-78-6	612	MS, RI	30.39 ± 1.52
3-Heptanone, 2-methyl-	13019-20-0	918	MS, RI	11.59 ± 0.58
Benzene, 1-ethenyl-2-methyl-	611-15-4	975	MS, RI	3.39 ± 0.17
d-Limonene	5989-27-5	1018	MS, RI	2.69 ± 0.13
Cyclohexane, pentyl-	4292-92-6	1135	MS, RI	16.80 ± 0.84
Phenylethyl Alcohol	60-12-8	1116	MS, RI	3.66 ± 0.18
Octanoic acid, ethyl ester	106-32-1	1196	MS, RI	2.66 ± 0.13
trans-1,2-Diethoxycyclohexane	93250-27-2	1192	MS, RI	2.50 ± 0.12
Cyclohexanone, cyclic trimethylene acetal	180-93-8	1212	MS, RI	4.41 ± 0.22
Oxalic acid, 2-ethylhexyl pentyl ester	1000309-38-7	1485	MS, RI	2.61 ± 0.13
Cyclohexanone, 2-cyclohexylidene-	1011-12-7	1551	MS, RI	7.08 ± 0.35
Hexadecanoic acid, ethyl ester	628-97-7	1993	MS, RI	15.41 ± 0.77
1,3,14,16-Nonadecatetraene	1000131-11-2	1924	MS, RI	12.96 ± 0.65
5.alpha.-Androstan-11-one, 19-hydroxy-	564-29-4	2183	MS, RI	14.53 ± 0.73
1H-Indole-2-carboxylic acid, 6-(4-ethoxyphenyl)-3-methyl-4-oxo-4,5,6,7-tetrahydro-, isopropyl ester	1000316-17-5	2823	MS, RI	5.49 ± 0.27

⁎ Ms is the mass spectrum; RI is the retention index.

In sample F, the volatile profile was dominated by hydrocarbons (*n* = 10), esters (*n* = 5), ketones (*n* = 3), alcohols (*n* = 2), and a diverse set of other volatiles (*n* = 13), including acids and phenolic derivatives. Prominent compounds included ethyl acetate (26.15 mg/L), 2-methyl-3-heptanone (194.33 mg/L), pentyl-cyclohexane (33.31 mg/L), tetradecane (28.71 mg/L), hexadecane (28.27 mg/L), hexadecanoic acid ethyl ester (58.55 mg/L), and oleic acid (34.41 mg/L). This wide distribution across volatile families contributed to a richer and more layered aroma profile, corroborating the high sensory scores for flavor complexity and acceptability.

By contrast, sample E exhibited a narrower spectrum with five hydrocarbons, five esters, three ketones, one alcohol, and one ether. The key volatiles included ethyl acetate (30.39 mg/L), pentyl-cyclohexane (16.80 mg/L), and hexadecanoic acid ethyl ester (15.41 mg/L). The reduced diversity and lower concentrations of volatiles in sample E align with the lower flavor complexity observed during sensory analysis.

Esters, particularly ethyl acetate and hexadecanoic acid ethyl ester, were prevalent in both samples and are well documented for their role in imparting fruity and floral notes in fermented plant-based matrices (Cvetković et al., 2020). Though less prominent in fruit beverages, hydrocarbons such as pentyl-cyclohexane were present at higher concentrations in sample F, potentially contributing green or waxy notes depending on compound-specific thresholds. The inclusion of ketones, especially 2-methyl-3-heptanone, further enriched sample F's aroma with buttery and floral nuances, consistent with previously reported contributions of ketones to volatile profiles in functional and fermented beverages (Kostyra et al., 2021).

The observed disparities in volatile content are attributed primarily to differences in ingredient ratios and matrix composition after blending. The elevated ester and ketone levels in sample F may be linked to its higher honey content and lower water proportion, which increased the concentration of fermentable sugars and aroma precursors. These factors likely enhanced the release and extraction of volatile compounds during analysis.

The higher aroma complexity observed in sample F is consistent with its enhanced phytochemical density. It aligns with previous findings that prolonged aging in vinegars—such as Chinese Shanxi or traditional barrel-aged types—leads to new volatile compounds and a more intense aroma profile (Callejón, Torija, Mas, Morales, & Troncoso, 2010).

This volatile analysis underscores the importance of optimizing ingredient combinations and fermentation parameters to develop vinegar-based beverages with improved functional and sensory attributes. For formulation development, the high ester and ketone levels in sample F serve as functional indicators of flavor enrichment, while the lower volatile content in sample E suggests opportunities for enhancement through ingredient adjustment or extended aging.

Therefore, sample F was selected for further computational bioactivity screening due to its superior performance across sensory, phytochemical, and volatile compound evaluations. These results reinforce the value of volatile profiling in standardizing aroma characteristics in fermented vinegar-based beverages.

### 3.6 *In silico* analysis results

The bioactive volatiles identified in the optimized formulation (F)—notably phenolic derivatives, fatty-acid–derived esters (*e.g.*, hexadecanoic acid ethyl ester), medium-chain ketones (*e.g.*, 2-methyl-3-heptanone), and long-chain hydrocarbons—are relevant not only to aroma but also to reported antioxidant and metabolic regulatory activities. Previous work on pomelo flower–green tea demonstrated that selected volatiles, including linalool, nerolidol, and indole, interacted with umami taste receptors through hydrogen bonding and hydrophobic forces, thereby linking volatile composition to receptor-level effects and enhanced taste perception (Ma et al., 2025).

Similarly, recent *in silico* investigations of phytosterols derived from medicinal mushrooms revealed strong binding affinities against monkeypox viral proteins, underscoring the broader applicability of docking approaches in evaluating the therapeutic potential of natural food-derived bioactives (Kaymak, Vural, & Çelik, 2025). In line with this evidence, we incorporated *in silico* docking and ADMET analyses as a computational component of the present study. This predictive approach provides insights into binding affinities, interaction mechanisms, and developability properties of selected volatiles with protein targets implicated in CVD and T2DM. By integrating this computational evaluation with sensory and compositional data, the results highlight the potential health relevance of the identified volatiles and help prioritize compounds for further biological validation.

**Fig. 5:**
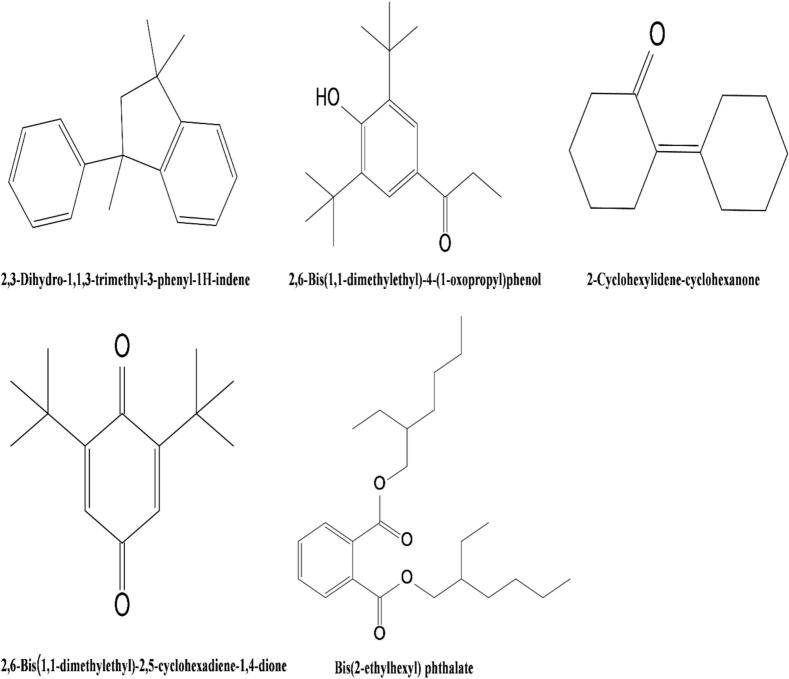
2D chemical structures of the top five compounds.

Molecular docking was conducted to evaluate the potential interactions between selected volatile compounds and protein targets implicated in CVD and T2DM. Based on the lowest binding energy values, the top five compounds are presented in Table 4, with their 2D chemical structures illustrated in Fig. 5.

**Table 4:** Docking analysis of the top five compounds with their corresponding lowest binding energy values.

CAS No.	Ligand Name	Binding Energy (Kcal/mol)			
		ACE	MAPK p38	SGLT1	1IR3
3910-35-8	2,3-Dihydro-1,1,3-trimethyl-3-phenyl-1*H*-indene	−8.4	−8.4	−8.2	−7.1
14035-34-8	2,6-Bis(1,1-dimethylethyl)-4-(1-oxopropyl)phenol	−7.2	−7	−7	−6.4
1011-12-7	2-Cyclohexylidene-cyclohexanone	−7	−6.7	−7.2	−6.4
117-81-7	Bis(2-ethylhexyl) phthalate	−6.7	−5.3	−7.3	−6.1
719-22-2	2,6-Bis(1,1-dimethylethyl)-2,5-cyclohexadiene-1,4-dione	−6.6	−6.2	−6.9	−6.3

**Fig. 6:**
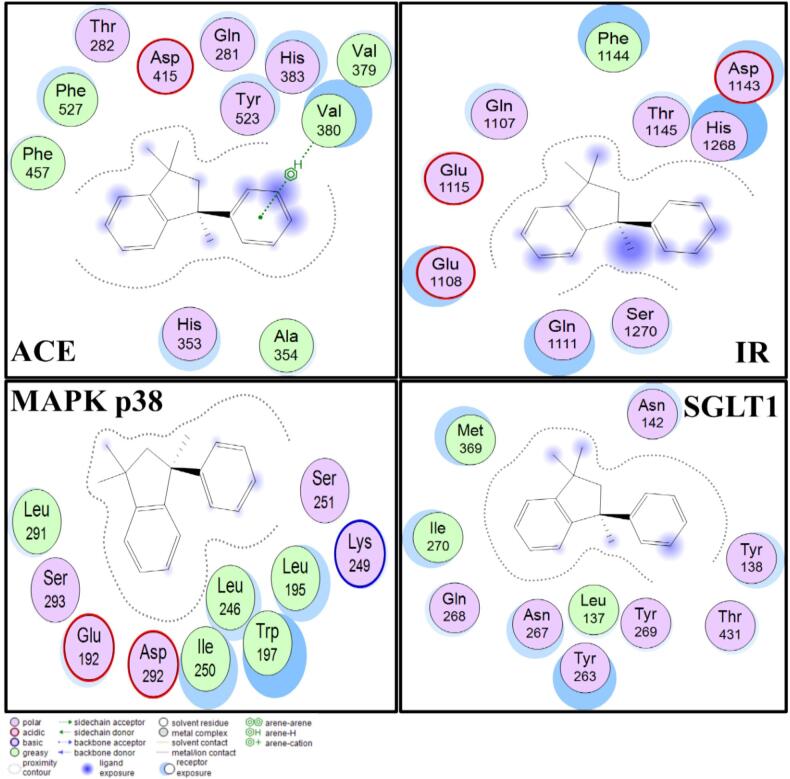
Binding interactions of the top compound, 2,3-Dihydro-1,1,3-trimethyl-3-phenyl-1H-indene (CAS #3910-35-8), with therapeutic target proteins visualized using the MoE tool. Critical residues identified include Gln281, Thr282, and His353 for ACE; Glu192 and Leu195 for MAPK p38; Gln1107 and Asp1143 for IR; and Leu137 and Tyr138 for SGLT1. These interactions may lead to potential therapeutics for CVD and T2DM.

Binding interactions of the most active compound, 2,3-dihydro-1,1,3-trimethyl-3-phenyl-1H-indene**,** were further visualized using Molecular Operating Environment (MoE) software (Fig. 6). Docking simulations revealed that 2,3-dihydro-1,1,3-trimethyl-3-phenyl-1H-indene displayed a strong binding affinity for the four selected protein targets: ACE, MAPK p38, IR, and sodium-SGLT1. Interaction with ACE involved key residues, including His353, Ala354, Phe457, and Tyr523, known to contribute to angiotensin II regulation and vasomodulation (Zheng et al., 2022). Binding to MAPK p38 involved Trp197, Ser251, and Asp292 residues, indicating possible influence on inflammatory cascades (Santhanakrishnan, Kalpana, Sathishkumar, Narasimhan, & Balakumaran, 2011).

The compound-bound IR at residues such as Gln1107, Asp1143, and His1268 for glucose metabolism targets, suggesting potential modulation of insulin signaling pathways (Murugan et al., 2024). Docking with SGLT1 revealed interactions with Tyr138, Asn267, and Gln268, indicating possible inhibition of renal glucose reabsorption and associated glycemic control mechanisms (Arif et al., 2021).

Among all tested volatiles, 2,3-dihydro-1,1,3-trimethyl-3-phenyl-1H-indene exhibited the strongest binding energies across ACE (−8.4 kcal/mol), MAPK p38 (−8.4 kcal/mol), SGLT1 (−8.2 kcal/mol), and IR (−7.1 kcal/mol), supporting its multi-target interaction potential. Importantly, 2,3-dihydro-1,1,3-trimethyl-3-phenyl-1H-indene has been previously reported in natural matrices, including sesame oil, tea leaves, and Hosta species (Dababneh, Protska, Kyslychenko, & Zhuravel, 2016), reinforcing its relevance as a plant-derived bioactive volatile.

#### 3.6.1 ADMET profiling results

**Fig. 7:**
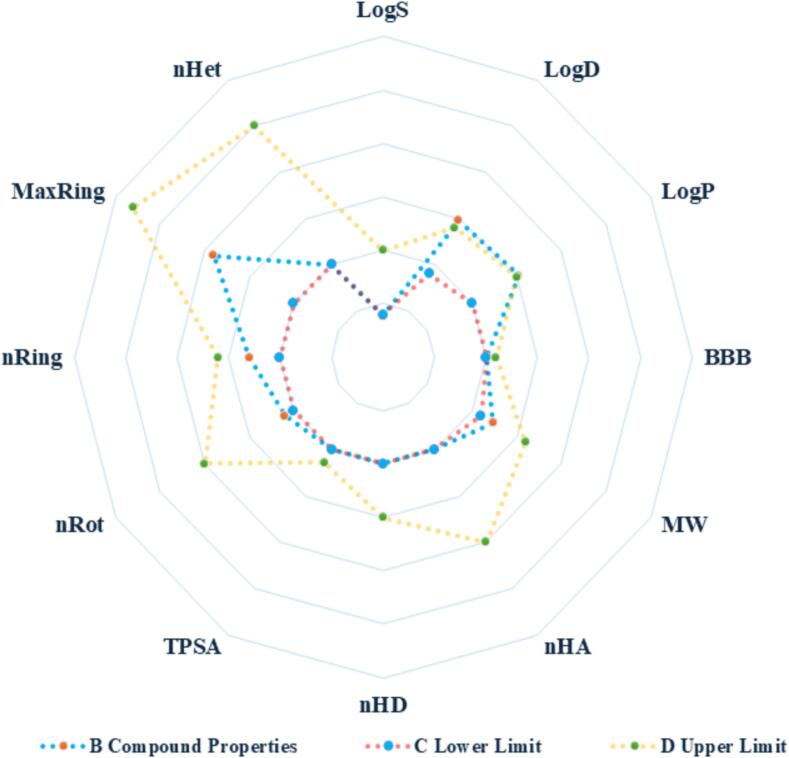
Radar plot showing the predicted ADMET profile of 2,3-dihydro-1,1,3-trimethyl-3-phenyl-1H-indene. The blue dotted line indicates predicted compound properties, while the red and yellow dotted lines represent the reference lower and upper limits, respectively. ADMET parameters include solubility (LogS), distribution (LogD, LogP), BBB penetration, molecular weight, hydrogen bond counts (nHA, nHD), TPSA, rotatable bonds (nRot), ring counts (nRing), and heteroatoms (nHet). (For interpretation of the references to color in this figure legend, the reader is referred to the web version of this article.)

Fig. 7 presents the ADMET profile of 2,3-dihydro-1,1,3-trimethyl-3-phenyl-1H-indene, with the blue dotted line representing the predicted values and the red and yellow dotted lines indicating the reference lower and upper limits, respectively.

The compound demonstrates favorable solubility (LogS), good lipophilicity (LogP), and efficient distribution (LogD), supporting its bioavailability and pharmacokinetic properties. It shows potential for crossing the blood-brain barrier (BBB) and has a moderate molecular weight (MW), making it ideal for drug-like properties. The hydrogen bond donors (nHD) and acceptors (nHA) suggest optimal interactions with biological targets, while the topological polar surface area (TPSA) and rotatable bonds (nRot) indicate favorable flexibility and permeability. The ring count (nRing) and heteroatom count (nHet) are within acceptable ranges, further supporting its chemical stability.

These favorable ADMET properties align with findings from recent studies on bioactive compounds derived from medicinal plants. For example, Merimi et al. (2025) demonstrated that flavonoids and rosmarinic acid exhibited favorable ADMET properties, such as high bioavailability, lipophilicity, and solubility, which are also observed in 2,3-dihydro-1,1,3-trimethyl-3-phenyl-1H-indene, supporting its therapeutic potential in cardiovascular diseases (CVD) and hypertension. Similarly, Flores-Holguín, Frau, and Glossman-Mitnik (2021) investigated Rosaceae plant cyclopeptides, highlighting their ADMET characteristics and suggesting their potential for drug development targeting metabolic disorders, which is consistent with our findings for 2,3-dihydro-1,1,3-trimethyl-3-phenyl-1H-indene.

These *in silico* predictions underscore the promising pharmacokinetic profile of 2,3-dihydro-1,1,3-trimethyl-3-phenyl-1H-indene, suggesting its potential as a lead compound for CVD and metabolic health therapies, pending further experimental validation to confirm these findings.

## 4 Conclusions

In conclusion, this study developed a value-added fermented beverage based on sun-aged red date vinegar enriched with goji berry juice and honey, balancing sensory appeal and potential health benefits. Sensory analysis and SPME–GC–MS profiling identified Sample F as the top formulation, characterized by a rich and complex volatile profile. Molecular docking further highlighted the possible bioactivity of key volatiles, particularly 2,3-dihydro-1,1,3-trimethyl-3-phenyl-1H-indene, as a candidate for interacting with targets related to cardiovascular disease and type 2 diabetes mellitus. These findings position sun-aged red date vinegar beverages as promising value-added products combining consumer acceptance with bioactive potential. To substantiate these outcomes, future *in vitro* and *in vivo* studies are necessary to verify the biological activity of the key compounds and determine their effective concentrations in physiological systems.

## CRediT authorship contribution statement

**Zeshan Ali:** Writing – review & editing, Writing – original draft, Supervision, Methodology, Data curation, Conceptualization. **Zinanone Rosaire Brottier:** Writing – original draft, Validation, Formal analysis, Data curation. **Jameel M. Al-Khayri:** Writing – review & editing. **Rana Adnan Tahir:** Writing – review & editing, Writing – original draft. **Sam Al-Dalali:** Writing – review & editing, Data curation. **Othman Al-Dossary:** Writing – review & editing. **Bader Alsubaie:** Writing – review & editing. **Mustafa I. Almaghasla:** Writing – review & editing.

## Funding

This study is supported by the Kunlun Mountain Elites Distinguished Education and Training Talent Studio (2025-01) and the Deanship of Scientific Research, Vice Presidency for Graduate Studies and Scientific Research, King Faisal University, Saudi Arabia [Grant No. KFU253231].

## Declaration of competing interest

The authors declare that they have no known competing financial interests or personal relationships that could have appeared to influence the work reported in this paper.

## Data Availability

Data will be made available on request.

## Appendix A Supplementary data

Supplementary material Figure S1. GC–MS total ion chromatogram (TIC) of volatile compounds in sample E. Figure S2. GC–MS total ion chromatogram (TIC) of volatile compounds in sample F. Supplementary material
