# Supplementary material for: Sun-aged red date vinegar-based beverage: integrated analysis of fermentation, sensory, volatile, and bioactive properties
Source: Food Chem X. 2025 Sep 12;31:103023. doi: 10.1016/j.fochx.2025.103023 (PMC12475855; doi:10.1016/j.fochx.2025.103023)
Supplement: Supplementary material — Figure S1. GC–MS total ion chromatogram (TIC) of volatile compounds in sample E. Figure S2. GC–MS total ion chromatogram (TIC) of volatile compounds in sample F. [file mmc1.docx]

**
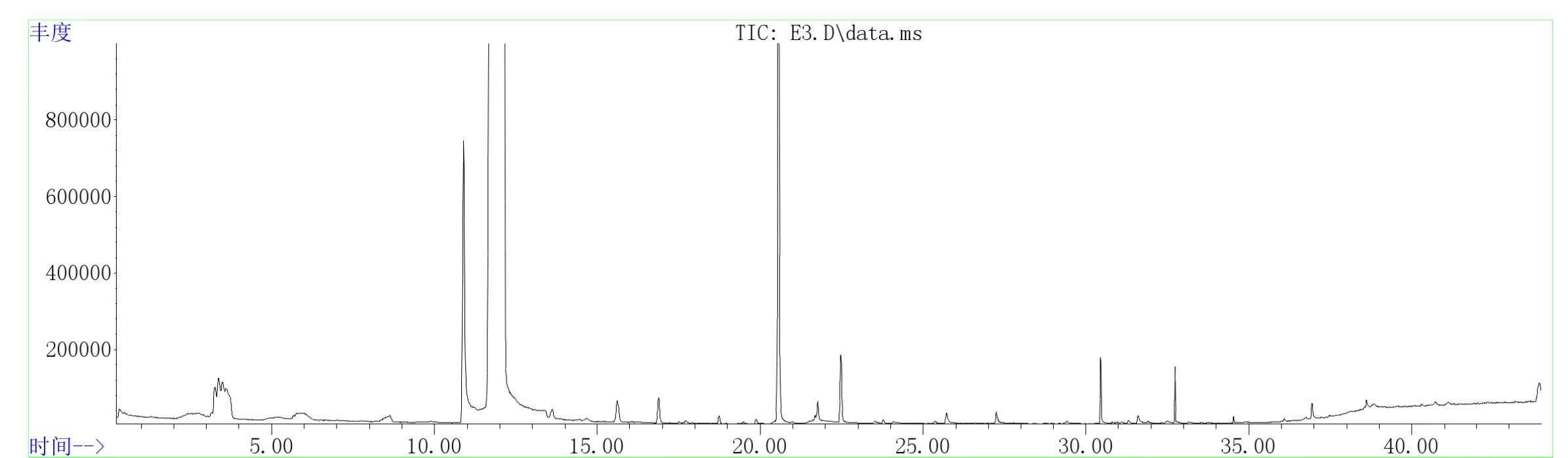
**

**Figure S1.** GC–MS total ion chromatogram (TIC) of volatile compounds in sample E.


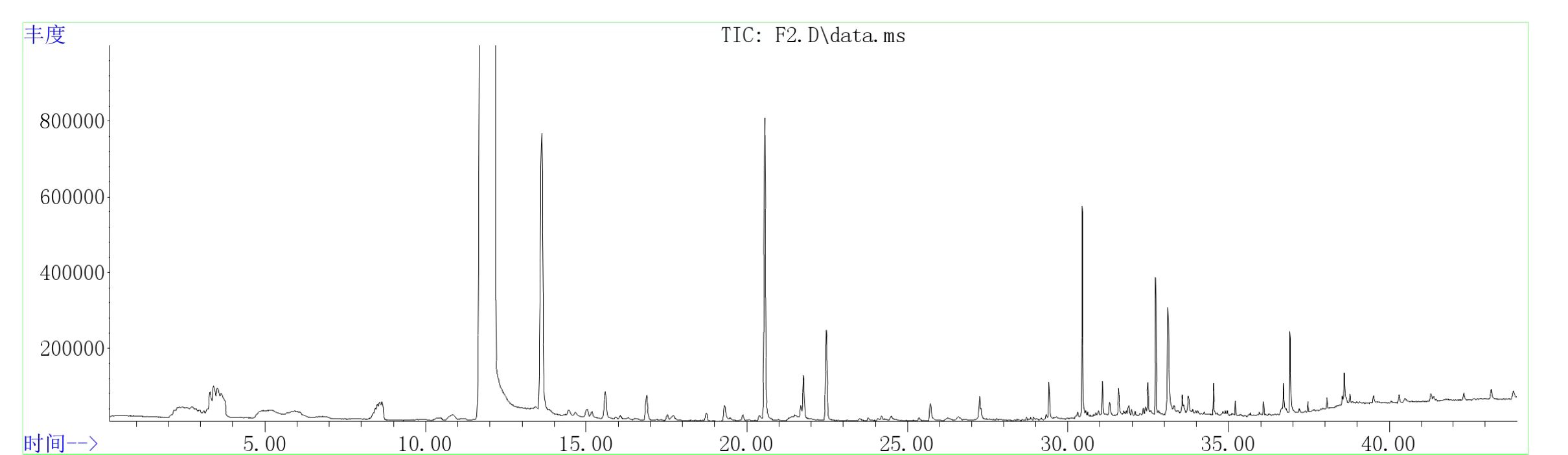


**Figure S2.** GC–MS total ion chromatogram (TIC) of volatile compounds in sample F.
